# Cost-effectiveness of an intensive group training protocol compared to physiotherapy guideline care for sub-acute and chronic low back pain: design of a randomised controlled trial with an economic evaluation. [ISRCTN45641649]

**DOI:** 10.1186/1471-2474-5-45

**Published:** 2004-11-23

**Authors:** Nicole van der Roer, Maurits W van Tulder, Johanna M Barendse, Willem van Mechelen, Willemien K Franken, Arjan C Ooms, Henrica CW de Vet

**Affiliations:** 1 Institute for Research in Extramural Medicine, VU University Medical Center, Amsterdam, The Netherlands; 2 Regional College for Physiotherapy Amsterdam, Amsterdam, The Netherlands; 3 Body@Work, Research Center Physical Activity, Work and Health, TNO-VUmc, Amsterdam, The Netherlands

## Abstract

**Background:**

Low back pain is a common disorder in western industrialised countries and the type of treatments for low back pain vary considerably.

**Methods:**

In a randomised controlled trial the cost-effectiveness and cost-utility of an intensive group training protocol versus physiotherapy guideline care for sub-acute and chronic low back pain patients is evaluated. Patients with back pain for longer than 6 weeks who are referred to physiotherapy care by their general practitioner or medical specialist are included in the study. The intensive group training protocol combines exercise therapy with principles of behavioural therapy ("graded activity") and back school. This training protocol is compared to physiotherapy care according to the recently published Low Back Pain Guidelines of the Royal Dutch College for Physiotherapy. Primary outcome measures are general improvement, pain intensity, functional status, work absenteeism and quality of life. The direct and indirect costs will be assessed using cost diaries. Patients will complete questionnaires at baseline and 6, 13, 26 and 52 weeks after randomisation.

**Discussion:**

No trials are yet available that have evaluated the effect of an intensive group training protocol including behavioural principles and back school in a primary physiotherapy care setting and no data on cost-effectiveness and cost-utility are available.

## Background

Low back pain is a very common complaint with major social and economical consequences. In a recent cross-sectional study the annual prevalence of low back pain in the general Dutch population was estimated at 44% [1]. The course of low back pain is usually relatively short: about 80–90% of people with low back pain spontaneously recover within four to six weeks. However, approximately 1–7% develop chronic low back pain. Although this is a relatively small group, the economic consequences are enormous [2]. The total costs of low back pain in the Netherlands in 1991 have been estimated at 1.7% of the Gross National Product [3]. About 93% of the total costs were due to absenteeism and disablement. Because of the enormous costs related to low back pain, effective interventions aimed at prevention and treatment of chronic complaints are necessary. The Cochrane Collaboration has published several systematic reviews on the effectiveness of different treatments for low back pain. Exercise therapy, back schools and behavioural therapy seem to be the most promising interventions for treatment of chronic low back pain [4]. Authors recommended future trials with sufficiently large sample sizes and sufficiently long follow-up periods. Cost-effectiveness and cost-utility analyses of treatments were also recommended, because the observed differences in effectiveness were only small.

Evidence-based physiotherapy for sub-acute and chronic low back pain patients consists of adequate information and an active approach, including behavioural principles. As physiotherapists have not yet put these principles into practice [5-7] two important barriers have to be dealt with. First, changing behaviour of health care providers is always very difficult, even when guidelines are actively implemented [8]. Second, physiotherapists usually do not have specific knowledge of behavioural principles and are usually not specifically trained to provide behavioural therapy. To solve these issues, physiotherapists in Amsterdam have developed a new intervention program. This program not only makes optimal use of the combination of the principles of exercise therapy, behavioural therapy and back schools, but has structured it into a protocol that facilitates physiotherapists to perform this intervention in clinical practice. This trial will evaluate the cost-effectiveness and cost-utility of the intensive group training protocol compared with physiotherapy guideline care.

## Methods

### Study design

The study is a randomised controlled trial (RCT). Alongside the trial a full economic evaluation will be conducted. The Medical Ethics Committee of VU University Medical Centre has approved the study design, protocols and informed consent procedures.

### Setting

The trial will be conducted in a primary physiotherapy care setting in Amsterdam and its surroundings. Eighty-five physiotherapists will participate in the trial; 40 physiotherapists are trained to provide the intensive group training protocol and 45 physiotherapist are instructed to provide usual physiotherapy care according to the Low Back Pain Guidelines of the Royal Dutch College for Physiotherapy (KNGF).

### Study population

Patients with non-specific low back pain referred to one of the participating physiotherapists by their general practitioner are eligible for participation in the trial. Patients are included if the current episode of low back pain lasts more than 6 weeks and if the complaints show no tendency to decrease, meaning that the patient has not increased his activities in the last three weeks. Furthermore, patients have to be between the age of 18 and 65 years old, live or work in Amsterdam and have a health insurance with one insurance company (Agis). This health insurance company covers about 80 to 90 percent of the Amsterdam population and is the only company that reimburses the intensive group training protocol. Patients are excluded from the study if 1) they have specific low back pain, attributable to e.g. infection, tumour, osteoporosis, rheumatoid arthritis, fracture, inflammatory process, radicular syndrome or cauda equina syndrome; 2) their general practitioner or medical specialist advised them not to perform physically straining activities; 3) they are pregnant; 4) they have pelvic pain/instability; 5) they are dealing with a lawsuit related to either their low back pain or related to their disability for work. Patients are recruited by participating physiotherapists. If patients are interested in participating in the trial, they receive written information about the trial and their name and phone number is given to a research assistant. The research assistant calls the patient two days later and explains the aim and implications of the study. If the patient agrees to participate, an appointment is made at a local research centre. At the local research centre a research physiotherapist checks again if the patients meets the eligibility criteria. Patients who meet the criteria and agree to participate in the trial must sign an informed consent form. Patients are asked to complete baseline questionnaires and the research physiotherapist will conduct baseline assessment of physiologic outcome measures.

In accordance with the CONSORT statement, information on number of recruited and eligible patients and reasons for exclusions or refusal to participate will be registered for all recruited patients by the participating physiotherapists and the research physiotherapist.

### Treatment allocation

Patients are randomly assigned to either the intensive group training protocol or physiotherapy guideline care. Randomisation is stratified for duration of complaints to ensure a sufficient number of sub-acute and chronic patients in each treatment group. To avoid inconvenience for the patients, seven local research centres are set up in different parts of the city. For each research centre two randomisation lists are prepared and permuted blocks of 4 patients are made to ensure equal distribution of patients for each research centre. An independent statistician generated the randomisation lists, using series of random numbers. The principle investigator (NvdR), who is not involved in the selection of patients, prepared the opaque, sealed envelopes, guaranteeing concealed randomisation. At the local research centre the administrative assistant hands the next envelope to the patient who then opens the envelope. The administrative assistant then checks the envelope and informs the participating physiotherapist about the treatment allocation.

### Blinding

Both the research physiotherapists and the principle investigator remain blinded for the allocation of treatment. Patients cannot be blinded for the interventions. As a consequence most outcome measures, consisting of self-report questionnaires are not blinded either. All physical outcome measures are blindly assessed by the research physiotherapist as we ask the patients not to reveal information about their treatment to the research physiotherapists. Participating physiotherapists can not be blinded for treatment allocation, but they are not involved in the assessment of outcome measurements.

### Interventions

Patients who are assigned to physiotherapy guideline care are treated according to the recently published Low Back Pain Guidelines of the Royal Dutch College for Physiotherapy (KNGF) [9]. The guidelines recommend giving adequate information, advising to stay active and providing exercise therapy with a behavioural approach for patients with sub-acute and chronic low back pain. As the guidelines are relatively new, physiotherapists providing the guideline care receive two training sessions of 2,5 hours each to ensure that the guidelines are properly applied. Preparation time of 2 hours before each session is strongly recommended. Two experts provide background information and discuss the content of the guideline. Video clips and statements on expected barriers are used to start discussions in groups of 10–15 physiotherapists supervised by expert trainers. After 4 months a follow-up session of 2,5 hours is organised to discuss practical problems and to ensure that all physiotherapists are working according to the guideline. The physiotherapists are asked to complete a form for each participating patient they treat, to register treatment goals, content of the treatment, total number of sessions in the treatment period and, if applicable, arguments to deviate from the guideline.

The intensive group training protocol combines exercise therapy with principles of back school and behavioural therapy. Back school principles include group lessons with adequate information on causes of low back pain, factors influencing low back pain, advice on physical activity and dealing with a relapse. Operant conditioning and graded activity as components of behavioural therapy are included in the protocol. Baseline measurements, goal setting and time-contingency are the main elements of the intervention. The purpose of the protocol is to improve activities and participation in work or other social activities, instead of focussing on pain or anatomical impairments. The patient has an active role and is responsible for the results of the therapy. The physiotherapist has the role of coach and focuses on the achieved improvement instead of the remaining complaints [10]. Active behaviour will be reinforced by the physiotherapist.

The protocol has a total duration of 30 weeks and consists of three phases: the starting phase, the treatment phase and the generalisation phase. It concerns 10 individual sessions of 30 minutes per session and 20 group sessions of 1,5 hours per session. During the first phase of three weeks, six individual sessions are planned for patient history and physical examination, providing information on the treatment, determining baseline level of functional capacity and signing a treatment contract. During the treatment phase the group sessions have a frequency of twice a week for eight weeks. Every patient has his own gradually increasing exercise program, with an operant-conditioning behavioural approach based on the baseline level of functional capacity. The treatment phase gradually changes into the generalisation phase in which patients learn to apply everything they have learned in the treatment phase to their own daily situation. Therefore the frequency of the sessions decrease in the last four weeks; patients are encouraged to exercise more at home and to choose a physical activity they will continue after treatment has finished. Two individual sessions are planned for evaluation during the twelve week training period and two additional individual sessions are planned three weeks and three months after the group sessions have finished.

The exercise program consists of:

1. warming-up and cooling down

2. aerobic exercises on a rowing machine, stationary bike or treadmill

3. muscle strengthening exercises of the lower back, abdomen and buttocks

4. exercises that specifically apply to the patient's situation

5. home exercises

The exercises mentioned at point 4. are determined by a Patient Main Complaint Form [11]. During the first intake patients are given this form, consisting of thirty different activities (e.g. turning in bed, lifting, walking, etc.). The patient is asked to select and prioritise the activities he has had trouble with during the last week and would very much like to see improved in the following months. The physiotherapist discusses the form with the patient and designs specific exercises for these activities. Three baseline measurements are performed to determine the maximal performance (for example, the maximum number of repetitions) for each exercise separately. The starting point of the program is 70% of the mean of all three measurements, in order to avoid failure and ensure the experience of success. In agreement with the patient the training quota are determined by the physiotherapist using the starting point, goals and training period to provide a gradually increasing program. The exercise goals are determined by the patient and physiotherapist together to ensure that goals are realistic, concrete, trainable and measurable. The treatment contract is signed by the patient and physiotherapist. The contract states that training quota are always followed exactly and that the patient keeps the graphs of finished sessions.

For training, the physiotherapists will receive instruction on the background and content of the protocol, and will be trained to include behavioural principles in the physiotherapeutic management of low back pain at two meetings of six hours each. In groups of 7–8 physiotherapists discussions and role playing are supervised by one expert trainer with extensive experience in behavioural principles. Four months after the last meeting 2 follow up sessions of 4 hours are organised to discuss practical problems and practice difficult situations with the trainers. The principal investigator (NvdR) will regularly visit the group sessions at the physical therapy practices to monitor the conduct of the intensive group training protocol. For each participating patient the physiotherapist is asked to complete a registration form containing the treatment goals, content of the different sessions and evaluation of the protocol.

### Contrast physiotherapy guideline care and intensive group training protocol

The intensive group training protocol is a standardised approach consisting of 30 treatment sessions. As the physiotherapy guideline care is not a protocol, the number of sessions will vary per patient. In daily practice the average number of treatment sessions is 9 and the average duration of treatment is 6 weeks [6]. The organisation of the intensive group training protocol is based on back school principles and will take place in groups of 5–8 patients. The physiotherapy guideline care is organised as usual physiotherapy care and patients are treated individually. The guidelines recommend exercise therapy with a behavioural approach. However, no further guidance is provided regarding the content of the exercise program (type, intensity, frequency and duration of exercises) or regarding integrating behavioural principles. In the intensive group training protocol the content of the exercise therapy, back school and operant condition are thoroughly described and the physiotherapists are trained to apply these skills in practice. So the contrast lies in the number of sessions, group versus individual therapy and the conduct of the behavioural therapy.

### Co-interventions and compliance

During the intervention period, co-interventions are discouraged. However, co-interventions will be reported and evaluated. Compliance to the intensive group training protocol is assessed by registering the number of treatment sessions that patients attend. The content of treatment and number of treatment sessions received by the physiotherapy guideline care group will be registered.

### Outcome assessment

In 1998 a proposal for standardised use of outcome measurement in low back pain studies was published [12]. An international group of investigators proposed a set of five domains that should be used in all low back pain studies: pain symptoms, back related function, general well being, disability and satisfaction with care. Additionally several other outcome measures that are commonly used in randomised trials in low back pain will be assessed.

#### Primary outcome measures

1. The functional status is assessed with the Roland Morris Disability Questionnaire [13]. The questionnaire consists of 24 questions related to activities of daily living. Each item is scored either 0 (disagree with statement) or 1 (agree with statement) and the total score ranges from 0 (no dysfunction) to 24 (maximum dysfunction).

2. General improvement is measured on a 6 point scale ranging from "much worse" to "completely recovered".

3. An 11-point numerical rating scale is used for determining pain intensity, ranging from 0 "no pain" to 10 "very severe pain" [14].

4. Work absenteeism is measured with the Short Form Health and Labour Questionnaire [15,16]. This questionnaire was developed for collecting quantitative data about the relation between illness, treatment and work-performance. Absence from work, reduced productivity at paid work, unpaid labour production and impediments to paid and unpaid labour are four dimensions that are addressed in the Health and Labour Questionnaire.

5. The EuroQol instrument is administered to assess the patient's general health status. The questionnaire describes the general health status in 5 dimensions: mobility, self-care, usual activities, pain/discomfort and anxiety/depression [17]. Because each of the five dimensions can be divided in 3 levels a total of 243 health states can be defined. Using the model by Dolan (1997) the total score will be expressed in utilities [18]. The official Dutch translation of the Euroqol will be administered.

#### Secondary outcome measures

6. The Tampa scale for kinesiophobia is developed as a measure of fear of movement/(re)injury by Miller et al. [19]. The questionnaire is relatively short and can easily be used in a primary care setting [20]. The scale consists of 17 items and each item is provided with a 4-point Likert scale ranging from "strongly disagree" to "strongly agree". The Dutch translation of the TSK by Vlaeyen et al. [21] will be used in the trial.

7. Cognitive and behavioural pain coping strategies are assessed using the Pain Coping Inventory [22]. This questionnaire consists of 6 factors: pain transformation, distraction, reducing demands, retreating, worrying and resting. All 34 items are scored on a four point scale where 1 equals "hardly ever/never" and 4 equals "very often". A recent validation study of the Pain Coping Inventory reported the coping scales to be reliable and sensitive enough to identify differences between coping strategies in pain patients [23].

8. Self-efficacy beliefs are measured using the Pain Self-Efficacy Questionnaire [24]. With the approval of Nicholas, the original 10-item questionnaire was translated into Dutch by the authors and subsequently translated back by a professional translator. Each item is scored on a 7-point scale ranging from 0 "not at all confident" to 6 "completely confident". By summarising the scores of all the items a total score is determined.

9. For measuring patient satisfaction four items (out of 17 items) of the Patient Satisfaction Scale of Cherkin et al. [25] are combined with nine items (out of 12 items) of the Patient Survey Instrument of Beattie et al. [26]. The Patient Satisfaction Scale was developed for measuring patient satisfaction with care they received from their physician and is a multidimensional disease specific measure, intended specifically for patients with low back pain. The Patient Survey Instrument is a multidimensional generic measure and was developed to determine the overall satisfaction with physical therapy. The items of the combined list are rated using a 5-point "agree-disagree" response format. The authors belief that the combination of both instruments is more applicable to the situation in the trial.

#### Physical measurements

The physical measurements will be performed at several local research centres. Therefore all physical tests must be easy to administer en practical. To minimize patient burden the tests should take as little time as possible en should not be too strenuous for the patient.

10. Anthropometric measurements will be done for interpretation of the physical outcome measures. Body weight, body height and skin fold measures are assessed. Skin fold thickness of biceps, triceps, subscapular and suprailiac will be assessed with a Harpenden skin fold calliper. The skin fold-thickness equation developed by Durnin and Womersley will be used to determine body fat mass [27].

11. Aerobic capacity will be assessed with the Chester Step Test [28,29]. This test was developed to determine the aerobic capacity in a relatively simple and practical way. The test is sub-maximal and ends when the heart rate of the participant reaches 75% of its predicted maximum. The test starts with a very slow step rate (15 steps per minute) and every two minutes the step rate increases with 5 steps per minute. Because the action of stepping is familiar to most people, the majority of the patients in the study will be able to perform the test.

12. The isometric endurance of the back muscles is evaluated with the test according to Ito [30]. The patient is positioned on the floor with a pillow under the abdomen and arms by the side. The patients raises the trunk to a horizontal position and the time the patient can maintain this position is measured.

13. The fingertop-to-floor distance is measured to determine the flexibility of the spine [31]. Standing with bare feet the participant will be asked to bend maximally forward with the feet together and the knees straight. The distance from the tips of the middle fingers to the floor is measured with a metal-ended tape measure.

#### Prognostic measures

At baseline, data of various prognostic measures will be collected to evaluate if randomisation successfully resulted in two prognostically comparable groups and to be able to adjust for baseline differences in the analysis, if necessary.

1. Data on individual factors such as age, gender, level of daily activity and preference for one of the treatment groups will be gathered by the administrative assistant.

2. Characteristics of low back pain: duration and severity of the current episode and number of previous episodes will be assessed by the research physiotherapists.

#### Cost data

The aim of the economic evaluation will be to determine and compare all back pain related costs of patients receiving the intensive group training protocol or physiotherapy guideline care. The costs will be related to the effects of the interventions. Cost effectiveness will be conducted from a societal perspective. Direct health care costs, including the costs for physiotherapy, additional visits to other health care providers, prescription medication, professional home-care and hospitalisation and direct non-healthcare costs such as out-of-pocket expenses, costs for paid and unpaid help and travel expenses will be included. Also data on indirect costs of loss of production due to back pain will be estimated for both paid and unpaid labour. Direct and indirect costs will be evaluated with cost diaries that patients keep during the whole time they participate in the trial [32]. The general health status is measured with the Dutch version of the EuroQol to compare the results of the cost-effectiveness analysis with other health care problems.

Patients will be asked to complete questionnaires at baseline and 6, 13, 26 and 52 weeks after randomisation. Physical measurements will be performed at baseline, 13 and 52 weeks after randomisation. Table 1 gives an overview of the data-collection.

### Sample size

To be able to detect a clinically relevant difference in pain intensity (improvement of 2 points on the 11-point pain intensity numerical rating scale after 52 weeks [33]) with a power (1-β) of 90% and a significance level of 5% (two-sided), two groups of 48 patients are needed. A population of chronic low back pain patients typically has a mean score of 7 (SD 2) on an 11-point pain intensity numerical rating scale. To be able to find a clinically relevant difference in disability (improvement of 3 points on the RDQ after 52 weeks [34]) with a power (1-β) of 90% and a significance level of 5%, two groups of 60 patients are needed. Chronic low back pain patients typically have a mean score of 15 (SD 5) on the RDQ.

We expect a drop-out rate of 10% at most. Drop-out rates of similar RCT's on neck pain and tennis elbow conducted at our institute were less than 3%. Therefore, to get complete data sets of 120 patients with sub-acute and 120 patients with chronic low back pain, 280 patients will be recruited, 140 sub-acute and 140 chronic low back pain patients. 85 Participating physiotherapists will be asked to recruit 5 patients; the recruitment period will be of 12 months duration.

### Statistical analysis

Intention-to-treat analyses will be conducted for all patients participating in both groups. A generalised linear mixed model will be applied to evaluate differences between groups over a period of 52 weeks. Subgroup analyses will be performed for duration of back pain: sub-acute (6–12 weeks) versus chronic (>12 weeks), for severity of complaints at baseline, for age, and for the psychosocial characteristics somatisation, fear avoidance, catastrophising and self efficacy.

Bootstrapping will be used for pair-wise comparison of the mean differences in direct health care, direct non-health care, total direct, indirect and total costs between the intervention groups. Confidence intervals will be obtained by bias corrected and accelerated (Bca) bootstrapping using 2000 replications [35]. Cost-effectiveness ratios will be calculated by dividing the difference between the mean costs of the two interventions by the difference in the mean effects of the two interventions. Ratios will include the primary clinical effect measures of the trial, i.e., general improvement, functional status, pain intensity and quality of life. Ratios will be graphically presented on a cost-effectiveness and cost-utility plane and acceptability curves will be calculated showing what the probability is that the intensive group training protocol is cost-effective at a specific ceiling ratio.

## Discussion

The intensive group training protocol includes interventions, such as exercise therapy, behavioural treatment and back school, that have recently been proven to be effective in patients with low back pain. The graded activity intervention is considered the be a form of behavioural treatment and earlier studies have proven the effectiveness of this intervention for workers who are sick-listed due to low back pain [36,37]. The trial by Staal et al. (2003) was conducted at an occupational health service department of an airline company in the Netherlands. In this study graded activity was found to be more effective than usual care in reducing the number of sick leave days. Lindström et al. (1992) examined the graded activity intervention in sick-listed workers at the Volvo factories in Sweden and showed a significant reduction in the number of days of sick leave. All participants in both studies were workers on sick leave due to low back pain. In a primary care setting the graded activity intervention has not yet been studied. Although participants in our study will follow an individual, gradually increasing exercise program, the training and back school will take place in a group setting.

In the Netherlands, the national physiotherapy guidelines for low back pain consist of general recommendations regarding diagnostic and therapeutic management of low back pain while the intensive group training protocol prescribes the frequency, intensity and duration of the exercise therapy, the content of informative group lessons and graded activity in detail. The intensive group training protocol is expected to be more effective, because it is a detailed protocol and because it combines principles of exercise therapy back school and behavioural therapy, which have recently been proven to be effective for this patient population in systematic Cochrane reviews [38-40].

Although the general practitioner, and if applicable the occupational physician, will be informed about the treatment and progress of the patient, the intervention is mono-disciplinary. A multidisciplinary intervention in a primary care setting has major practical implications and would increase the costs of the intervention considerably. The intensive group training protocol itself probably generates higher costs than physiotherapy guideline care but we expect reduction in health care utilization and productivity losses in the long term, compensating for the increase in treatment cost. This trial will provide physiotherapists with more knowledge and experience in behavioural treatment for low back pain patients and may increase the efficiency of physiotherapeutic care for this complex and expensive patient group. If the intensive group training protocol appears to be more cost-effective than physiotherapy guideline care, a future update of the national physiotherapy guideline will include more specific recommendations in line with this protocol. In that case the protocol will be implemented throughout the Netherlands.

## List of abbreviations

KNGF = Royal Dutch College for Physiotherapy

Bca bootstrapping = bias corrected and accelerated bootstrapping

## Competing interests

The author(s) declare that they have no competing interests.

## Author's contributions

NvdR is responsible for the data collection and drafted the manuscript. MWvT, JMB, WvM and HCWdV were involved in developing the original idea for funding and were co-applicants on the successful funding proposal. WKF and ACO both will contribute to data collection and processing. All authors participated in development of research protocols and in the design of the study. All authors read and corrected draft versions of the manuscript.

## Pre-publication history

The pre-publication history for this paper can be accessed here:



## Figures and Tables

**Table 1:** Overview of data collection

Outcome measures	Follow-up				
	t = 0	t = 6	t = 13	t = 26	t = 52

**Primary outcomes**					
Functional status (RDQ)	X	X	X	X	X
General improvement		X	X	X	X
Pain intensity	X	X	X	X	X
Work absenteeism (SF-HLQ)	X	X	X	X	X
General health (Euroqol)	X	X	X	X	X
**Secondary outcomes**					
Fear avoidance (Tampa)	X	X	X	X	X
Coping (PCI)	X	X	X	X	X
Self-efficacy	X	X	X	X	X
Patient satisfaction		X	X	X	X
Aerobic capacity	X		X		X
Flexibility	X		X		X
Strength	X		X		X
Height	X				
Weight	X		X		X
Skinfold measurements	X		X		X
**Other**					
General (age, gender, education, back pain episodes)	X				
Cost diaries	X	X	X	X	X
